# Examination of Impact of After-Hours Admissions on Hospital Resource Use, Patient Outcomes, and Costs

**DOI:** 10.1155/2022/4815734

**Published:** 2022-11-23

**Authors:** Charlenn Skead, Laura H. Thompson, Hanna Kuk, Ariel Hendin, Moosa Yasir Hamood Al Abri, Yasmeen Choudhri, Tim Ramsay, Brent Herritt, Kwadwo Kyeremanteng

**Affiliations:** ^1^Department of Medicine, University of Ottawa, Ottawa, Ontario, Canada; ^2^Ottawa Hospital Research Institute, Ottawa, Ontario, Canada; ^3^Department of Emergency Medicine, University of Ottawa, Ottawa, Ontario, Canada; ^4^Division of Critical Care, Department of Medicine, University of Ottawa, Ottawa, Ontario, Canada; ^5^Department of Critical Care Medicine, Comox Valley Hospital, Courtenay, Canada; ^6^Division of Palliative Care, Department of Medicine, University of Ottawa, Ottawa, Ontario, Canada

## Abstract

**Background:**

Nighttime and weekends in hospital and intensive care unit (ICU) contexts are thought to present a greater risk for adverse events than daytime admissions. Although some studies exist comparing admission time with patient outcomes, the results are contradictory. No studies currently exist comparing costs with the time of admission. We investigated the differences in-hospital mortality, ICU length of stay, ICU mortality, and cost between daytime and nighttime admissions.

**Methods:**

All adult patients (≥18 years of age) admitted to a large academic medical-surgical ICU between 2011 and 2015 were included. Admission cohorts were defined as daytime (8:00–16:59) or nighttime (17:00–07:59). Student's *t*-tests and chi-squared tests were used to test for associations between days spent in the ICU, days on mechanical ventilation, comorbidities, diagnoses, and cohort membership. Regression analysis was used to test for associations between patient and hospitalization characteristics and in-hospital mortality and total ICU costs.

**Results:**

The majority of admissions occurred during nighttime hours (69.5%) with no difference in the overall Elixhauser comorbidity score between groups (*p*=0.22). Overall ICU length of stay was 7.96 days for daytime admissions compared to 7.07 days (*p*=0.001) for patients admitted during nighttime hours. Overall mortality was significantly higher in daytime admissions (22.5% vs 20.6, *p*=0.012); however, ICU mortality was not different. The average MODS was 2.9 with those admitted during the daytime having a significantly higher MODS (3.0, *p*=0.046). Total ICU cost was significantly higher for daytime admissions (*p*=0.003). Adjusted ICU mortality was similar in both groups despite an increased rate of adverse events for nighttime admissions. Daytime admissions were associated with increased cost. There was no difference in all hospital total cost or all hospital direct cost between groups. These findings are likely due to the higher severity of illness in daytime admissions.

**Conclusion:**

Daytime admissions were associated with a higher severity of illness, mortality rate, and ICU cost. To further account for the effect of staffing differences during off-hours, it may be beneficial to compare weekday and weeknight admission times with associated mortality rates.

## 1. Introduction

The hours immediately following hospital and intensive care unit (ICU) admission are crucial for influencing patient outcomes since vulnerability to complications is greatest at this time [1–3]. In this context, it is important that diagnosis and treatment plan formulation occur rapidly to reduce patient mortality, regardless of the time of admission [4, 5]. Nights and weekends (off-hours) have been recognized as high-risk admission times for both hospital and ICU patients [4–6]. Typically, off-hours consist of fewer staff on-call, with a greater proportion of these staff being less experienced compared to daytime staff [7]. Few studies exist which examine the relationship between admission time and patient outcomes. Furthermore, existing study results are contradictory, perhaps because of differences across study sites in terms of patient characteristics and staffing [7–9]. Studies conducted in Saudi Arabia, the United Kingdom, and Taiwan show that including more experienced intensivists and more detailed management guidelines during off-hours improved ICU patient outcomes on weekends and weeknights [4, 10, 11]. However, these studies tend not to account for differences in illness severity or resources needed. Multiple studies have found no difference in mortality, with some studies showing a decrease in mortality rates for night time admissions [1, 5, 8, 11–17]. For studies analyzing admission times for single illnesses or injuries such as hip fractures or gastrointestinal haemorrhage, the results generally show significant differences in patient outcomes between regular hours and off-hours, with patients admitted during off-hours having higher mortality rates [18, 19].

We are not aware of studies comparing costs between daytime and nighttime admissions. However, it is an important question as it allows for further investigation into more cost-effective practice for daytime and nighttime ICU admissions.

The objective of this study was to investigate the mortality rates between daytime and nighttime admissions as well as patient outcomes such as ICU length of stay, days on mechanical ventilation, the occurrence of adverse events, and cost.

## 2. Methods

Setting and patient collection: This retrospective cohort study included all adult patients (≥18 years of age) admitted to an ICU at The Ottawa Hospital between January 1, 2011, and December 31, 2015. Patients were followed up for the length of their hospitalization until discharge or up to one year, whichever came first, including transfers out of and back into the ICU if applicable. Patients were classified according to the first initial ICU admission time as daytime (08:00–16:59) or nighttime (17:00–07:59) admission cohorts, correlating with the presence or lack of an attending presence, respectively.

Data Collection: All data were obtained from the Ottawa Hospital Data Warehouse, a health administrative database that integrates several information resources including a clinical data repository and case-costing system [20, 21]. Baseline patient demographic data, previous admission, transfer data, and patient diagnosis at the time of ICU admission were assessed in parallel for the daytime and nighttime patient cohorts. Comorbid conditions were summarized and presented as the Elixhauser (ELIX) comorbidity index that uses coded information based on the International Classification of Diseases, Version 10 (ICD-10-CA) [22, 23]. Outcome data were collected from the time of admission until either the point of discharge from the hospital or death and included ICU length of stay, discharge data, and patient safety indicators, among others. Further, hospital costs accrued by patients including total hospital costs, direct costs, and costs of an ICU stay were assessed [21]. Total hospital costs included both direct and indirect costs. Direct costs refer to all expenses paid to the hospital with fee codes linked to the patient chart. This includes salaries and benefits for the management staff, equipment, screening, and materials but does not include physician's remuneration due to the billing structure within the province. Indirect costs refer to any overhead operational fees including the cost of the room occupied by the patient on an hourly basis. Case costing at the Ottawa Hospital is determined based on the Ontario case costing initiative [24] described in the Canadian Institute for Health Information Management Information Systems Guidelines [25]. All costs were reported in Canadian dollars (CDN).

Data Analysis: Statistical analyses were performed using GraphPad Prism Software v. 8.3.0 (538) and Stata v. 11. Continuous variables including age, ELIX score, days spent in ICU, and days on mechanical ventilation were compared between daytime and nighttime admission cohorts using the Student's *t*-test (parametric values) with a level of significance of a *p* value of equal to or less than 0.05. This data was presented as a calculated mean value followed by the standard deviation (SD). Patient data pertaining to cost analysis, including total ICU costs and total hospital-associated costs (including direct costs) were assessed using a Student's *t*-test and presented as mean values followed by standard deviation (SD) values. Chi-square tests (*χ*^2^) were used for the remaining variables to determine the differences among patients between the daytime and nighttime patient cohorts. *p* value equal or less than 0.05 was considered statistically significant. Regression analysis was used to test for associations between patient age, gender, the Elixhauser Comorbidity Score, the Multiorgan Dysfunction Score (MODS), use of dialysis, mechanical ventilation, and the most responsible diagnosis (MRD) and (1) in-hospital mortality and (2) total ICU costs. MRD was captured by the ICD-10 code. Missing data for ICU interventions accounted for 18.6% of patients. The analysis that included these variables was done with complete data only.

This study was approved by the Ottawa Health Science Network Research Ethics Board.

## 3. Results

During the study period, 14,265 patients were admitted to the ICU. Patient characteristics are summarized in Table 1. The average age was 63.74, and 56% of patients were male, with no statistical difference between daytime versus nighttime admissions.

Less than one third of admissions (30.5%) occurred during daytime hours (08:00–16:59) with 69.5% occurring during nighttime hours (17:00–07:59) (Table 1). Patients were significantly more likely to be admitted directly from home (21% vs 13.8%, *p* < 0.0001) or from a surgical ward (48.6% vs 46.9%, *p* < 0.0001) when admitted during daytime hours (Table 1). Patients were also significantly more likely to be transferred from other institutions during the daytime (37.3% vs 32.8%, *p* < 0.001). The majority of MRD fell under “other” (39.3%)—which included diseases of the digestive system, infectious and parasitic diseases, and neoplasms—followed by disease of the circulatory system (33.3%) (Table 1). A significantly higher number of admissions for respiratory disease occurred at night (12.4 vs 10.9%, *p* < 0.001) as well as “other” (40.4% vs 36.6%, *p* < 0.001). There was no difference in the overall Elixhauser comorbidity score between groups (Table 1). However, significantly more patients with valvular disease, peripheral vascular disease, COPD, and liver disease were admitted during nighttime hours. The average MODS for all admitted patients was 2.9 with those admitted during the daytime having a significantly higher MODS (3.0, *p*=0.046) than those admitted during the nighttime hours.

The comparison of patient outcomes by admission period is shown in Table 2. The need for mechanical ventilation was similar in both groups; however, daytime admissions had a significantly longer duration of invasive mechanical ventilation (3.8 vs 3.2, *p* < 0.001). More daytime admitted patients required dialysis (9.8% vs 8.7%, *p*=0.035). The overall ICU length of stay was 8.0 days for daytime admitted patients compared to 7.1 days for patients admitted during nighttime hours (*p* < 0.001). Daytime admissions were more likely to be transferred to acute-care inpatient institutions (14.3% vs 12.5%) or transferred to continuing care (13.5% vs 12.1%) upon ICU discharge.

In-hospital mortality was significantly higher in daytime admissions (22.5% vs 20.6, *p*=0.012); however, ICU mortality showed no difference (Table 2). A significantly higher number of adverse events occurred in the nighttime admissions group (36.5% vs 32%, *p* < 0.001) with a higher amount of cardiac (8.5% vs 7.2%, *p*=0.01) and surgical complications (6.1% vs 4%, *p* < 0.001) (Table 2). There was no significant difference in drug-related adverse events, traumatic injuries arising in hospital, hospital-acquired infections, or death rate in low-risk patients.

In the multivariable analysis, greater age, female gender, higher Elixhauser comorbidity scores, certain most responsible diagnoses (digestive, infectious, and respiratory), higher MODS, and mechanical ventilation were associated with higher odds of in-hospital mortality (Table 3). In a similar multivariable analysis, higher Elixhauser comorbidity scores, certain most responsible diagnoses (infectious and respiratory), dialysis, and mechanical ventilation were associated with higher total ICU costs (Table 3). Greater age was associated with lower total ICU costs in this multivariable analysis. Time of admission was not found to be associated with in-hospital mortality (Table 3).

The total ICU cost was significantly higher in those admitted during the daytime (*p*=0.0027) (Table 4). However, there was no difference in all hospital total cost or all hospital direct cost between groups. The adjusted total ICU cost for time of admission was similar for both groups (Table 3).

## 4. Discussion

The first hours in the ICU have a large influence on patient outcomes [1–3]. Previous studies have noted that off-hour admissions carry higher risks for patients [4–6]. To our knowledge, no previous studies have compared costs between daytime and nighttime admissions. In this single-center, retrospective cohort study of a mixed ICU, we found the majority of admissions occurred during nighttime hours. A number of studies have reported more admissions occurring during nighttime hours [8, 10, 11, 14, 15]. This, however, varies, with some studies showing higher admissions during the day [1, 6, 12, 26]. Overall, the Elixhauser score was similar between groups. Previous studies have shown that patients admitted during off-hours including nighttime and weekends had higher severity of illness scores; however, scores used differed between studies [13, 16, 26–28]. Luyt 2007 found that patients admitted during the day had a higher severity of illness. This is in agreement with our study which demonstrated that those admitted during the daytime had a higher severity of illness as evidenced by their higher MODS. This is also in keeping with the overall crude mortality being significantly higher for those admitted during the day, compared to those admitted during nighttime hours (*p*=0.012). Conflicting results have been reported for mortality associated with time of admission. The majority of studies demonstrated no difference between daytime and nighttime admission mortality rates [1, 5, 8, 11–14]. Two meta-analyses [4, 7] supported this. Laupland 2008 showed a decrease in mortality for daytime admissions, and other studies showed increased mortality with after-hour admissions [8, 26, 28, 29].

There was no difference in odds of ICU mortality or cost between groups. This is reassuring, as the majority of academic ICUs including ours do not have a board-certified intensivist in house during nighttime hours, only residents and/or fellows. In other studies, no difference in mortality rate was found for off-hour admissions with 24/7 staff on-site [10] or with a staff intensivist on call [17, 30, 31].

Numerous factors likely play into this. Firstly, a board-certified intensivist is available on call 24/7, and patients admitted during off-hours are usually reviewed in person with a fellow or staff member within 8 hours. Sicker patients also tended to be admitted during the daytime, which allows for staff intensivist input. The nursing ratio is the same during day and night and services such as endoscopy, consultants, and other procedures are available during off-hours. During the day, numerous obligations require time from ICU staff and residents such as teaching, discharging, procedures, family updates, and team rounds that are not generally required at night. Typically, more testing and invasive procedures are ordered during the daytime, which are associated with their own morbidity and mortality [15].

The main concern in assessing the difference between day and nighttime admissions is that patients admitted at night with fewer staff and no intensivist in-house may be at an increased risk of adverse outcomes. This study demonstrates that nighttime admissions are not associated with increased odds of morality. However, an overall higher rate of complications occurred at night, specifically, a higher amount of cardiac and surgical complications. Surgical complications may be related to fewer surgical staff and more emergency surgeries occurring at night. Kerlin et al. found that a higher rate of intraoperative adverse events and postoperative pulmonary complications occurred with surgeries occurring at night. This is consistent with our results which demonstrated a higher rate of surgical complications at night, with 77% of all surgical complications occurring at night [33]. Studies have also demonstrated that even with only trainees in the ICU overnight, patient outcomes are not improved, and the number of adverse events does not decrease by having more physicians or more senior physicians in the ICU during the night [33–35].

While the higher severity of illness as evidenced by higher MODS, days of mechanical ventilation, requirement for dialysis, and length of stay is likely contributing to the higher in-hospital mortality rate for daytime admissions, other factors may also be contributing. For example, daytime allows for admission from elective surgery, during which complications may arise, and a significantly higher rate of transfers from the surgical ward occurred during the day in our study. Secondly, a higher number of transfers from other institutions occurred during the day and those who were admitted during the day were also more likely to require transfer to other acute care institutions or continuing care, pointing to the overall severity of illness. It may also be that due to logistical factors that admissions to the ICU during the day are delayed, and delayed admissions have been shown to increase mortality rates [4, 36].

While previous studies have assessed the difference in mortality and patient outcomes between daytime and nighttime admissions, none, to our knowledge, have addressed possible cost differences; however, with rising costs of healthcare, this is an important parameter to assess and may have potential practice-changing implications. Our study found that those admitted during the daytime had a significantly higher total ICU cost. The adjusted ICU cost was similar between groups. This is likely due to the lower severity of illness for nighttime admissions. There was no difference between nighttime and daytime admission for overall hospital cost. This is likely explained by the increased severity of illness and mortality rates in those admitted during the daytime, therefore not requiring a prolonged hospital stay. This study has limitations including that it is a retrospective single-institution study along with the use of multiple comparisons. It did not compare medical versus surgical patients, as we have mixed ICUs. Secondly, intensivists were present during the day on weekdays and weekends, so it may have been beneficial to assess the mortality rate comparing weekdays vs weekends and/or weekday nights vs weekend nights to further assess the impact of the presence of a qualified intensivist. Physician remuneration was not included in this study which may have altered the cost analysis.

## 5. Conclusion

Overall, we found that nighttime ICU admission is not associated with worse outcomes or higher mortality rates. Protective factors include the availability of procedures and consultants at all times, ICU staff on call 24/7, and the use of early ICU assessment through our ICU outreach rapid response team.

## Figures and Tables

**Table 1:** Patient characteristics by admission time period.

Variable name	Overall *n* *=* 14,265 (100%)	Daytime hours (08:00–16:59) *n* *=* 4,352 (30.5%)	Nighttime hours (17:00–07:59) *n* *=* 9,913 (69.5%)	*p* value
Age at admission, years, mean (SD)	**63.74 (17.07)**	**63.35 (17.22)**	**63.91 (17.00)**	0.076
Gender, male sex, *n* (%)	**8,061 (56.5%)**	**2,419 (55.6%)**	**5,642 (56.9%)**	0.140
Previous location before first ICU, *n* (%)				
Direct admission (home)	2,284 (16.0)	914 (21.0)	1,370 (13.8)	**<0.001**
Emergency department	6,765 (47.4)	2,113 (48.6)	4,652 (46.9)	0.074
Surgical ward	952 (6.7)	63 (1.4)	889 (9.0)	**<0.001**
Medical ward	4,264 (29.9)	1,262 (29.0)	3,002 (30.3)	0.123
Transfer from other institutions, *n* (%)	**4875 (34.2)**	**1622 (37.3)**	**3253 (32.8)**	**<0.001**
Elixhauser comorbidities, *n* (%)				
Congestive heart failure	1,805 (12.7)	566 (13.0)	1,239 (12.5)	0.402
Arrhythmia	2,377 (16.7)	713 (16.4)	1,664 (16.8)	0.552
Valvular disease	330 (2.3)	123 (2.8)	207 (2.1)	**0.007**
Peripheral vascular disease	1,104 (7.7)	218 (5.0)	886 (8.9)	**<0.001**
Hypertension, total	4,357 (30.5)	1,345 (30.9)	3,012 (30.4)	0.534
Chronic obstructive pulmonary disease	1,470 (10.3)	485 (11.1)	985 (9.9)	**0.029**
Diabetes mellitus, total	4,239 (29.7)	1,316 (30.2)	2,923 (29.5)	0.365
Renal failure	713 (5.0)	232 (5.3)	481 (4.9)	0.227
Liver disease	610 (4.3)	164 (3.8)	446 (4.5)	**0.047**
Metastatic cancer	854 (6.0)	253 (5.8)	601 (6.1)	0.563
Haematological malignancy	334 (2.3)	113 (2.6)	221 (2.2)	0.182
Elixhauser comorbidity score, mean (SD)	**5.35 (6.3)**	**5.2 (6.1)**	**5.3 (6.3)**	0.223
Average MODS, mean (SD)	**3.9 (2.9)**	**4.0 (3.0)**	**3.8 (2.9)**	**0.046**
Most responsible diagnosis (ICD diagnosis), *n* (%)				
Diseases of the respiratory system	1,558 (10.9)	539 (12.4)	1,019 (10.3)	**<0.001**
Diseases of the circulatory system	4,753 (33.3)	1,482 (34.1)	3,271 (33.0)	0.218
Diseases of the nervous system	391 (2.7)	133 (3.1)	258 (2.6)	0.127
Mental and behavioural disorders	98 (0.7)	21 (0.5)	77 (0.8)	0.050
Injury, poisoning, and other external causes	1,863 (13.1)	584 (13.4)	1,279 (12.9)	0.399
Other	5,602 (39.3)	1,593 (36.6)	4,009 (40.4)	**<0.001**

**Table 2:** Patient outcomes by admission time period.

Variable name	Overall *n* = 14,265 (100%)	Daytime hours (08:00–16:59) *n* = 4,352 (30.3%)	Nighttime hours (17:00–07:59) *n* = 9,913 (69.5%)	*p* value
Invasive mechanical ventilation, *n* (%)	6,293 (44.1)	1,967 (45.2)	4,326 (43.6)	0.084
Dialysis, *n* (%)	1,284 (9.0)	425 (9.8)	859 (8.7)	0.035
ICU LOS days, mean (SD)	7.3 (11.6)	8.0 (14.1)	7.1 (10.2)	<0.001
Duration of invasive mechanical ventilation, days, mean (SD)	3.4 (7.)	3.8 (9.0)	3.2 (6.2)	<0.001
Mortality, total, *n* (%)	3,021 (21.2)	978 (22.5)	2,043 (20.6)	0.012
Mortality at ICU, *n* (%)	2570 (18.0)	836 (19.2)	1734 (17.5)	0.662
Discharged home with no support services, *n* (%)	5,094 (35.7)	1,493 (34.3)	3,601 (36.3)	0.020
Discharged home, support services required, *n* (%)	2,326 (16.3)	613 (14.1)	1,713 (17.3)	<0.001
Transferred to an acute care inpatient institution, *n* (%)	1,856 (13.0)	617 (14.2)	1,239 (12.5)	0.006
Transferred to continuing care, *n* (%)	1,787 (12.5)	588 (13.5)	1,199 (12.1)	0.019
Patient safety indicators, *n* (%)				
Any adverse event	5,005 (35.1)	1,391 (32.0)	3,614 (36.5)	<0.001
Cardiac complication	1160 (8.1)	315 (7.2)	845 (8.5)	0.010
Drug-related adverse event	193 (1.4)	57 (1.3)	136 (1.4)	0.767
Traumatic injury arising in hospital	45 (0.3)	13 (0.3)	32 (0.3)	0.813
Hospital acquired infection	2,279 (16.0)	666 (15.3)	1,613 (16.3)	0.146
Surgical complication	779 (5.5)	176 (4.0)	603 (6.1)	<0.001
Death in low risk patients	96 (0.7)	33 (0.8)	63 (0.6)	0.409

**Table 3:** Multiple regression analysis between time of admission and mortality and total ICU costs.

Factors	In-hospital mortality		Total ICU costs	
	OR (95% CI)	*p* value	*β*coefficient (95% CI)	*p* value
Age	1.03 (1.03–1.03)	*p* < 0.0001	−71.99 (−120.93–−23.05)	*p*=0.004
Gender				
Male	REF			
Female	1.13 (1.03–1.24)	*p*=0.011	−337.62 (−1942.5–1267.25)	*p*=0.68
Elixhauser score	1.05 (1.04–1.05)	*p* < 0.0001	320.05 (186.81–453.28)	*p* < 0.0001
Time of admission				
0800–1659	REF		REF	
1700–0759	0.94 (0.85–1.04)	*p*=0.220	−1508.06 (−3234.73–218.60)	*p*=0.087
Most responsible diagnosis				
Circulatory	REF			
Digestive	1.29 (1.08–1.55)	*p*=0.005	2372.13 (−801.45–5545.71)	*p*=0.143
Infectious	1.58 (1.34–1.86)	*p* < 0.0001	10044.43 (7105.94–12982.92)	*p* < 0.0001
Injury/poisoning	0.96 (0.81–1.13)	*p*=0.596	977.39 (−1724.40–3679.19)	*p*=0.478
Neoplasm	1.11 (0.91–1.36)	*p*=0.315	2695.91 (−812.66–6204.49)	*p*=0.132
Other	0.94 (0.81–1.09)	*p*=0.426	−2275.01 (−4705.19–155.17)	*p*=0.067
Respiratory	1.47 (1.25–1.73)	*p* < 0.0001	12314.44 (9479.94–15148.94)	*p* < 0.0001
MODS	1.23 (1.20–1.25)	*p* < 0.0001	−262.81 (−611.48–85.85)	*p*=0.140
Dialysis				
No	REF			
Yes	0.89 (0.77–1.02)	*p*=0.098	28243.37 (25596.64–30890.10)	*p* < 0.0001
Invasive mechanical ventilation				
No	REF			
Yes	1.93 (1.72–2.17)	*p* < 0.0001	21641.66 (19701.09–23582.24)	*p* < 0.0001

**Table 4:** Costs by admission time period.

Variable name	Overall *n* *=* 14,265	Daytime hours (08:00–16:59) *n* *=* 4,352	Nighttime hours (17:00–07:59) *n* *=* 9,913	*p* value
Total ICU cost, mean (SD)	27,170 (43,163)	28,809 (49,945)	26,450 (39,805)	**0.003**
All hospital total cost, mean (SD)	39,038 (60,133)	40,389 (67,934)	38,445 (56,363)	0.076
All hospital direct cost, mean (SD)	29,440 (45,497)	30,444 (51,387)	28,999 (42,651)	0.081

## Data Availability

The data used to support the findings of this study are available from the corresponding author upon request.

## References

[1] Meynaar I. A., van der Spoel J. I., Rommes J. H., van Spreuwel-Verheijen M., Bosman R. J., Spronk P. E. (2009). Off hour admission to an intensivist-led ICU is not associated with increased mortality. *Critical Care*.

[2] Rivers E., Nguyen B., Havstad S. (2001). Early goal-directed therapy in the treatment of severe sepsis and septic shock. *New England Journal of Medicine*.

[3] Wood K. E. (2002). Major pulmonary embolism. *Chest*.

[4] Galloway M., Hegarty A., McGill S., Arulkumaran N., Brett S. J., Harrison D. (2018). The effect of ICU out-of-hours admission on mortality. *Critical Care Medicine*.

[5] Uusaro A., Kari A., Ruokonen E. (2003). The effects of ICU admission and discharge times on mortality in Finland. *Intensive Care Medicine*.

[6] Vest-Hansen B., Riis A. H., Sorensen H. T., Christiansen C. F. (2015). Out-of-hours and weekend admissions to Danish medical departments: admission rates and 30-day mortality for 20 common medical conditions. *BMJ Open*.

[7] Cavallazzi R., Marik P. E., Hirani A., Pachinburavan M., Vasu T. S., Leiby B. E. (2010). Association between time of admission to the ICU and mortality. *Chest*.

[8] Brunot V., Landreau L., Corne P. (2016). Mortality associated with night and weekend admissions to ICU with on-site intensivist coverage: results of a nine-year cohort study (2006-2014). *PLoS One*.

[9] McDowell M. M., Kellner C. P., Sussman E. S. (2014). The role of admission timing in the outcome of intracerebral hemorrhage patients at a specialized stroke center. *Neurological Research*.

[10] Arabi Y., Alshimemeri A., Taher S. (2006). Weekend and weeknight admissions have the same outcome of weekday admissions to an intensive care unit with onsite intensivist coverage. *Critical Care Medicine*.

[11] Sheu C. C., Tsai J. R., Hung J. Y. (2007). Admission time and outcomes of patients in A medical intensive care unit. *The Kaohsiung Journal of Medical Sciences*.

[12] Wunsch H., Mapstone J., Brady T., Hanks R., Rowan K. (2004). Hospital mortality associated with day and time of admission to intensive care units. *Intensive Care Medicine*.

[13] Barnett M. J., Kaboli P. J., Sirio C. A., Rosenthal G. E. (2002). Day of the week of intensive care admission and patient outcomes: a multisite regional evaluation. *Medical Care*.

[14] Laupland K. B., Shahpori R., Kirkpatrick A. W., Stelfox H. T. (2008). Hospital mortality among adults admitted to and discharged from intensive care on weekends and evenings. *Journal of Critical Care*.

[15] Luyt C. E., Combes A., Aegerter P. (2007). Mortality among patients admitted to intensive care units during weekday day shifts compared with “off” hours. *Critical Care Medicine*.

[16] Morales I. J., Peters S. G., Afessa B. (2003). Hospital mortality rate and length of stay in patients admitted at night to the intensive care unit. *Critical Care Medicine*.

[17] Numa A., Williams G., Awad J., Duffy B. (2008). After-hours admissions are not associated with increased risk-adjusted mortality in pediatric intensive care. *Intensive Care Medicine*.

[18] Kristiansen N. S., Kristensen P. K., Nørgård B. M., Mainz J., Johnsen S. P. (2016). Off-hours admission and quality of hip fracture care: a nationwide cohort study of performance measures and 30-day mortality. *International Journal for Quality in Health Care*.

[19] Xia X. F., Chiu P. W. Y., Tsoi K. K. F., Chan F. K. L., Sung J. J. Y., Lau J. Y. W. (2018). The effect of off-hours hospital admission on mortality and clinical outcomes for patients with upper gastrointestinal hemorrhage: a systematic review and meta-analysis of 20 cohorts. *United European Gastroenterology Journal*.

[20] Ronksley P. E., McKay J. A., Kobewka D. M., Mulpuru S., Forster A. J. (2015). Patterns of health care use in a high-cost inpatient population in Ottawa, Ontario: a retrospective observational study. *CMAJ Open*.

[21] Fernando S. M., Rochwerg B., Reardon P. M. (2018). Emergency department disposition decisions and associated mortality and costs in ICU patients with suspected infection. *Critical Care*.

[22] Van Walraven C., Austin P. C., Jennings A., Quan H., Forster A. J. (2009). A modification of the Elixhauser comorbidity measures into a point system for hospital death using administrative data. *Medical Care*.

[23] Elixhauser A., Steiner C., Harris D. R., Coffey R. M. (1998). Comorbidity measures for use with administrative data. *Medical Care*.

[24] Ministry of Health and Long-Term Care (2013). *Ontario Case Costing Guide*.

[25] Canadian Institute for Health Information (2011). *Canadian Patient Cost Database Technical Document: MIS Patient Costing Methodology*.

[26] Bhonagiri D., Pilcher D. V., Bailey M. J. (2011). Increased mortality associated with after-hours and weekend admission to the intensive care unit: a retrospective analysis. *Medical Journal of Australia*.

[27] Ensminger S. A., Morales I. J., Peters S. G. (2004). The hospital mortality of patients admitted to the ICU on weekends. *Chest*.

[28] Kuijsten H. A. J. M., Brinkman S., Meynaar I. A. (2010). Hospital mortality is associated with ICU admission time. *Intensive Care Medicine*.

[29] Abella A., Enciso V., Torrejón I. (2016). Effect upon mortality of the extension to holidays and weekends of the ‘“ ICU without walls ”’ project . A before-after study. *Medicina Intensiva*.

[30] Kerlin M. P., Small D. S., Cooney E. (2013). A randomized trial of nighttime physician staffing in an intensive care unit. *New England Journal of Medicine*.

[31] Wilcox M. E., Chong C. A. K. Y., Niven D. J. (2013). Do intensivist staffing patterns influence hospital mortality following ICU admission? A systematic review and meta-analyses. *Critical Care Medicine*.

[32] Cortegiani A., Gregoretti C., Neto A. S. (2019). Association between night-time surgery and occurrence of intraoperative adverse events and postoperative pulmonary complications. *British Journal of Anaesthesia*.

[33] Kerlin M. P., Small D. S., Cooney E. (2013). A randomized trial of nighttime physician staffing in an intensive care unit. *New England Journal of Medicine*.

[34] Kerlin M. P., Harhay M. O., Kahn J. M., Halpern S. D. (2015). Nighttime intensivist staffing, mortality, and limits on life support: a retrospective cohort study. *Chest*.

[35] Wallace D. J., Angus D. C., Barnato A. E., Kramer A. A., Kahn J. M. (2012). Nighttime intensivist staffing and mortality among critically ill patients. *New England Journal of Medicine*.

[36] Cardoso L. T. Q., Grion C. M. C., Matsuo T. (2011). Impact of delayed admission to intensive care units on mortality of critically ill patients: a cohort study. *Critical Care*.

